# Italian Response to Coronavirus Pandemic in Dental Care Access: The DeCADE Study

**DOI:** 10.3390/ijerph17196977

**Published:** 2020-09-24

**Authors:** Luca Aquilanti, Silvia Gallegati, Valerio Temperini, Luigi Ferrante, Edlira Skrami, Maurizio Procaccini, Giorgio Rappelli

**Affiliations:** 1 Department of Clinical Specialistic and Dental Sciences, Polytechnic University of Marche, 60126 Ancona, Italy; l.aquilanti@pm.univpm.it (L.A.); m.procaccini@staff.univpm.it (M.P.); g.rappelli@staff.univpm.it (G.R.); 2 Department of Management, Polytechnic University of Marche, 60121 Ancona, Italy; v.temperini@staff.univpm.it; 3 Centre of Epidemiology, Biostatistics and Information Technology, Polytechnic University of Marche, 60126 Ancona, Italy; l.ferrante@staff.univpm.it

**Keywords:** COVID-19, coronavirus, public opinion, dental care access, behavior, survey, consumer perception, anxiety

## Abstract

The aim of the study was to evaluate the impact of the coronavirus pandemic on the willingness, anxiety and concerns of Italian people on undergoing dental appointments. An anonymous survey was posted online on social media on 11 May 2020 and was completed by 1003 respondents in one week. Multiple correspondence analysis and multiple logistic regression were used to evaluate the association between socio-demographic characteristics, dental care access, contagion fear of severe acute respiratory syndrome coronavirus-2 (SARS-CoV-2), trust in dentists regarding sanitization procedures and perception of the impact of the risk of contagion on dental care. Subjects with a high level of education, attending public dental offices and that are used to go to dental offices for urgent care would not feel comfortable in undergoing a dental appointment and would prefer to postpone or cancel dental visits, waiting for a decrease in the number of the contagions. Moreover, the risk of canceling or postponing the appointment at the dentist was 1.59 times greater in those who claimed to be strongly influenced by SARS-CoV-2. Fear of coronavirus disease (COVID-19), new cases decrease and the not urgent nature of dental visits influenced more than the lowered income household on upcoming or resuming dental appointments. In the next months, despite the forecasted economic crisis caused by coronavirus pandemic, fear and anxiety generated by the spread of the virus will impact more than the lowered familiar income with regards to access to dental care.

## 1. Introduction

Coronavirus disease (COVID-19) is an infectious disease caused by a novel coronavirus: SARS-CoV-2 (severe acute respiratory syndrome coronavirus-2) [1]. The first case of COVID-19 was reported in Wuhan (Hubei, China) on 31 December 2019. Rapidly, the virus has spread worldwide. On 30 January 2020, the World Health Organization (WHO) declared the outbreak of a global health emergency. On March 11th, 2020, WHO announced a pandemic [2].

According to the Italian Health Ministry, to date more than 248,000 cases were diagnosed with COVID-19 and more than 35,000 deaths due to such infection were recorded [3]. Italy has been hit hard by the rapid and disruptive diffusion of SARS-CoV-2. Italian Prime Minister Giuseppe Conte imposed a national quarantine and lockdown starting March 9th, 2020, aimed at restricting movements of the population except for health reasons, work and undelayable necessities, in order to contrast the growing number of COVID-19 cases in the country [4,5].

The coronavirus pandemic has rapidly changed health conditions, social relationships, economic prospects and daily routine life around the world. Lucchese and Pianta [6] forecast a severe financial and economic crisis related to coronavirus pandemic, highlighting the need to understand the extent of the crisis itself and the social, health and economic challenges that are linked to it. Beside the possible worsening of financial and economic income of Italian families due to the spread of coronavirus, the feelings generated by the pandemic could also play a role in changing daily life and social relationships. COVID-19 has reached great public attention, via both mass and social media, and a huge amount of information about the virus and its outbreak have been reported and searched [7,8]. 

Even though Italian dental offices have never stopped working, they limited their practice ensuring just urgent and emergency dental care treatments. Droplet production and blood and saliva exposure put dental practitioners at high risk of contagion during their routine procedures [9,10,11]. Aerosol or droplets inhalation from SARS-CoV-2 positive individuals and even the direct contact with mucous membranes, oral fluids and contaminated instruments and surfaces could enhance virus transmission during dental procedures [12,13]. The strict measures adopted in Phase 1 were progressively loosened starting on May 4th, 2020, when Italy, following the Decree of the President of the Council of Ministries, entered so-called Phase 2 [14]. Most of the productive activities returned to operativity even if measures such as social distancing remained strict. From May 4th, 2020 onward, it was once again possible for the Italian people to access dental care for non-urgent reasons. Thus, the DeCADE (DEntal Care Access During Emergency) study was aimed at assessing the feelings of the Italian population, their concerns and willingness to undergo or resume dental visits during and after the period of quarantine imposed by the Sars-CoV-2 pandemic.

## 2. Materials and Methods

A questionnaire was created using the free-access platform powered by Google (https://forms.gle/sAP8E5D3pWgs9pn46). Data collection took place in the time period between May 11th and May 18th, 2020. The questionnaire was submitted in the very early moments of Phase 2 in order to describe feelings and concerns of Italian people during Phase 1 of pandemic and to assess the impact these could have on scheduling or resuming dental visits during and after the period of quarantine imposed by the Sars-CoV-2 pandemic.

A self-administrated standard anonymous questionnaire was posted on Facebook, on the private accounts of two members of the research group (L.A. and S.G.). Facebook was chosen as a preferred social media for the purposes of the research because of its increasing relevance as a social network, as a communication tool for the users and also for the significant amount of time that users spend on the platform [15]. A snowball sampling technique was used. The sampling frame for the present research was taken from the Facebook’s friends list of two of the researchers: using the snowball approach, all the respondents were asked to share the link with other people. 

The questionnaire, in Italian, was composed of 43 questions, divided into three sections (Supplementary Material Table S1). The first section recorded socio-demographic data (age, gender, education, employment, etc.) and aimed at assessing the first economic impacts brought by the coronavirus (effects of Sars-CoV-2 on the household income) (from question number 1 to question number 8, Supplementary Material Table S1). The second section was aimed at analyzing the relationship of each individual with coronavirus: fear of being infected, channels used to gather information, reliability of the communication channels, perspectives on future activities of the individual after the end of the pandemic (from question number 9 to question number 23, Supplementary Material Table S1). Finally, the purpose of the third and last section was to investigate the habits of individuals with concern to access to oral care (from question number 24 to question number 43, Supplementary Material Table S1). Respondents were asked question about their experiences at the dentist and future plans to access oral care with the purpose of understanding if the coronavirus pandemic had affected the availability and willingness of Italian people to undergo oral care treatments. Multiple choice questions (question number: 1, 2, 4, 5, 6, 7, 8, 12, 15, 20, 21, 22, 23, 24, 26, 27, 28, 29, 31, 32, 33, 35, 36, 42, 43), open answer question (question number: 3), 1 to 5 rating scale questions (question number: 9, 10, 11, 13, 16, 17, 18, 19, 37, 38, 39, 40), where 1 corresponded to Not at all and 5 to Extremely, and dichotomous Yes/No questions (question number: 14, 25, 30, 34, 41) were used in the present questionnaire (Table S1). Moreover, variables were further classified and used for the analysis as reported in Supplementary Material Table S2.

### Statistical Analysis

A descriptive analysis was performed to evaluate the main characteristics of the participants in the study. Absolute and percentage frequencies were calculated to summarize the characteristics collected. 

Multiple correspondence analysis (MCA) was used to explore the association between socio- demographic characteristics, dental care access, the fear of SARS-CoV-2 contagion during Phase 1, the approach with dental care visit during Phase 2, the perception of the impact of the risk of contagion on dental care and trust in dentists regarding sanitization procedures, were considered altogether. MCA enables the identification of associations among variables when considered simultaneously. The modalities of the categorical variables are organized in a multiple contingency table, and a low-dimensional explanation for the interaction or the dependence between rows and columns of the table is provided. This analysis allows a geometrical representation of the results by locating each variable modality as a point on the Cartesian axes according to the frequency of the modalities: closer points indicate that the corresponding modalities are shared by the subjects, and modalities are considered associated. Results are interpreted according to the relative position of the points and consist of looking for groupings of variables modalities as those with the closer distance between points. The association between variables can be described by several identified dimensions and the number of the axes depends on the variability explained by each dimension.

A multiple logistic regression was carried out to estimate the independent effect of the fear related to the pandemic and the behavior individuals would have during Phase 2 in approaching new or scheduled dental visits on the probability of the perceived impact of COVID-19 pandemic on modifying the behavior on the upcoming dentist appointments. The analysis was adjusted for socio-demographic characteristics of the individuals.

All statistical analyses were performed with R statistical software [16] and the threshold of significance was set at 5%.

## 3. Results

Overall, 1003 respondents completed the survey within the 7 days of the publication of the questionnaire. Out of the total of respondents, 60.7% was female and 39.3% was male. The largest share of respondents, 32.4%, belonged to the age group 25–34, followed by those aged between 45 and 54, who made up the 18.1% of the sample; furthermore, 17.6% was aged 18–24, 15.7% was between 35 and 44 years old, 12.2% was between 55 and 64 years old, 2.6% was between 65 and 74 years old and 1.4% was more than 75 years old. Most of the respondents came from Central Italy (Lazio, Marche, Tuscany, Umbria), with a total share 48.0%, 38.1% from South Italy (Abruzzo, Calabria, Campania, Molise, Apulia, Sardinia, Sicily), and 11.9% from North Italy (Emilia Romagna, Friuli Venezia Giulia, Liguria, Lombardy, Piedmont, Trentino-South Tyrol, Veneto,). Table 1 shows the sociodemographic data of the participants.

Table 2 showed the answers of respondents regarding their habits concerning their access to dental care and their attitude toward resuming or undergoing dental visits. In total, 61.2% of participants declared they are used to go to dental offices for regular visits and professional dental hygiene sessions, 27.5% declared they only go for emergencies and 8.6% stated they are under a dental treatment plan. Furthermore, 2.7% of the sample (*n* = 27) indicated that they had never been to a dentist. Among those participants who were used to accessing dental care (*n* = 976), 93.2% of them declared they attend private dental offices. Only 6.8% of the sample declared to attend either public dental structures or low-cost dental offices or private dental offices affiliated to the national public health system. In total, 71.6% of the respondents declared to have attended the same dentist for at least three years, 16% for two or three years, and 12.4% for less or equal than one year.

The second part of the third section of the survey was dedicated to those who needed dental treatments during the period from 9th March 2020 to 4th May 2020, the so-called Phase 1, when access to dental visits was only allowed for emergency reasons. In total, 9.4% (*n* = 94) of the respondents underwent dental treatment during the Italian lockdown period. Among those who underwent dental treatment, all considered, 82.8% reported a positive experience, 10.8% did not know how to judge the treatment and 4.2% reported a negative experience. 

The third part of the third section of the survey was aimed at investigating how participants would have approached or had approached dental care appointments, considering the lockdown Phase 2. In total, 24.2% of the respondents declared they already had a scheduled dental visit in the upcoming few months. Considering all the answers, 59.1% of the participants thought they would not have any problem in undergoing the dental appointment, 22.6% would perform dental cares with Sars-CoV-2 fear, 16.9% would like to postpone their appointment and 1.4% would prefer to cancel their dental appointment. The respondents were also asked about the main reason to cancel a dental appointment. Out of 1003 participants, 59.7% declared that the main reason to drop dental visits was the non-urgency of the treatment, 15.1% indicated fear of contagion from coronavirus as the main responsible, 8.9% answered that the lowered economic income was the cause of dental appointment cancellation and 1.7% indicated other reasons.

The fourth and last part of section three of the questionnaire reflected the sense of trust and the communication between the dentist and the patient. In total, 82.4% of the sample reported a high sense of trust in the dentist (values of 4 and 5 in a 1 to 5 scale), while 17.6% reported a low level of trust (values of 1, 2 and 3 in a 1 to 5 scale). Among the participants of the present survey, a share of 44.1% was informed about all the procedures that were followed in order to sanitize the dental office, mostly through a phone call (33.5%), followed by a talk with the dentist (13.6%) and social media communication (9.1%). In total, 51.1% of the respondents declared they would call the dental office or the dentist directly in order to receive further information, while 37.3% of the participants would not ask for supplemental details.

**Figure 1:**
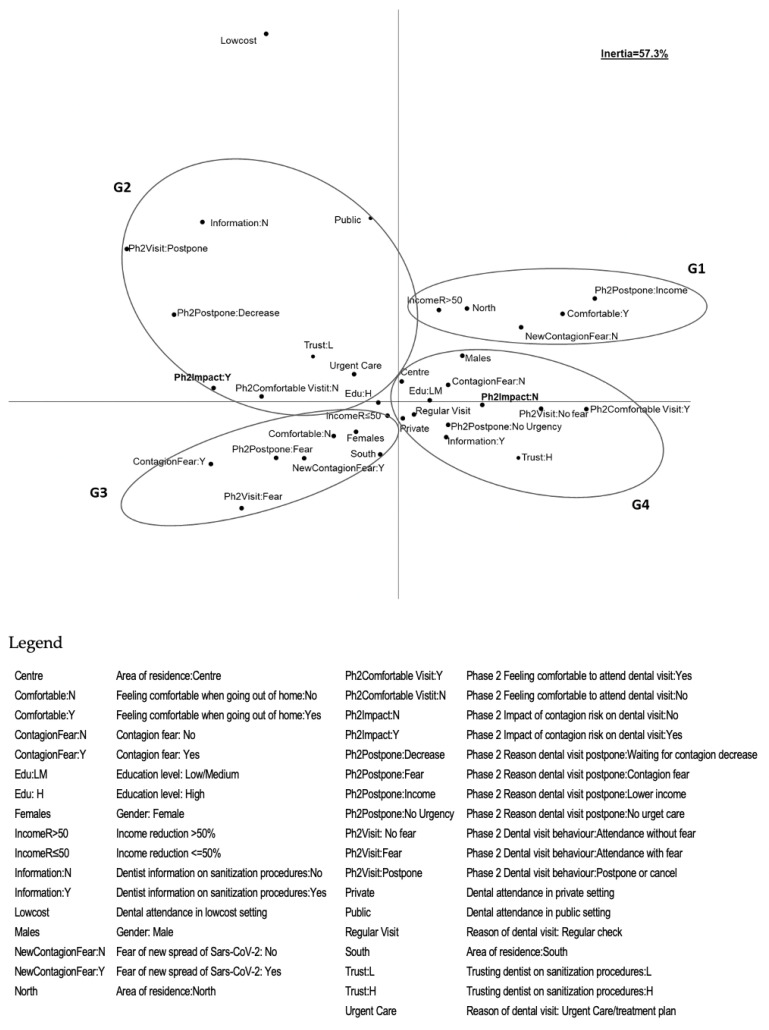
Association between socio-demographic characteristics, dental care access, the fear of severe acute respiratory syndrome coronavirus-2 (SARS-CoV-2) contagion during Phase 1, the approach with dental care visit during Phase 2, the perception of the impact of the risk of contagion on dental care and trust in dentists regarding sanitization procedures. Results of the Multiple Correspondence Analysis (MCA): briefly, G1 group is characterized by subjects coming from North Italy that are not afraid of a new spread of the virus; G2 group identifies subjects with high level of education that would not feel comfortable in undergoing a dental appointment; G3 group refers to subjects coming from South Italy that would postpone a dental appointment; G4 group includes subjects with low or medium level of education that would feel comfortable in undergoing a dental appointment.

Results of the MCA are shown in Figure 1. The two dimensions detected explained about 57% of the total variability (52% and 5% by the first and second dimension, respectively). The following main groups of associated variable modalities were identified:

- The group on the top right (G1) was characterized by subjects living in North Italy, whose income was reduced over 50% due to the pandemic. They feel comfortable going out of their home, are not afraid of a new spread of the virus and would postpone the dental visit for economic reasons
- The group placed on the top left of the graph (G2) identified subjects with a high level of education, attending public dental offices and that are used to go to dental offices for urgent care. During Phase 2 they would not feel comfortable in undergoing a dental appointment and would prefer to postpone or cancel dental visits, waiting for a decrease in the number of the contagions. Moreover, they did not receive all the information on dentist’s sanitization procedures, had a low level of trust with regard to these procedures and had the perception that the risk of contagion would have an impact on their upcoming dental visits
- The group on the bottom left (G3) referred to subjects living in South Italy, mostly females, with a reduction in income due to the pandemic of less than 50%. They fear SARS-CoV-2 contagion, do not feel comfortable getting out of their home and fear a new spread of the virus. They fear dental visits during Phase 2 and would postpone because of fear of getting infected
- The last group on the bottom right of the graph (G4) mostly included men with low or medium levels of education, living in regions of Central Italy, usually attending private dental offices for regular visits. They do not fear contagion and would not have any problem with and would feel comfortable in undergoing a dental appointment during Phase 2; moreover, they would postpone dental visits in case of non-urgent need for treatment. They had received information on all the procedures that were followed in order to sanitize the dental office, having a high sense of trust in the dentist and having the perception that SARS-CoV-2 would have no impact on their dental visits.

Table 3 shows the associations among sociodemographic characteristics, fear of COVID-19, respondents’ Phase 2 behavior, dental office attendance and the influence of SARS-CoV-2 on upcoming dental appointments. The multiple logistic regression showed that the risk of canceling or postponing the appointment at the dentist was 1.59 times greater in those who claimed to be strongly influenced by SARS-CoV-2. Fear of COVID-19, new cases decrease and the not-urgent nature of dental visits had a higher influence on the lowered income household with regards to upcoming or resuming dental appointments (3.12, 3.55 and 2.23, respectively). Those who attended either private or public dental offices were, respectively, 0.21 and 0.15 times less influenced than those who used to go to low-cost chains. People with low/medium educational level were 0.67 times less influenced by SARS-CoV-2 than those with high education. 

## 4. Discussion

Several surveys have been proposed since the spread of SARS-CoV-2 pandemic in order to assess the impact of coronavirus on dental activities, highlighting the great psychological distress among dental staff [17,18,19,20]. Nevertheless, it could be considered the fact that such questionnaires were delivered during a very sensitive moment of the pandemic, in which severe levels of anxiety could be found worldwide. To the best of our knowledge, the present study was the first that aimed to assess the feelings of the Italian population and their response to SARS-CoV-2 in undergoing or resuming dental treatment during and after the period of quarantine. Only one study tried to assess the level of anxiety and concerns of orthodontic patients regarding the coronavirus pandemic and the impact of the quarantine in appointments and in their orthodontic treatment, showing that the quarantine and coronavirus pandemic impacted orthodontic appointments and patients’ anxiety [21].

Natural pandemics, principally those with high rates of infection and mortality, are often responsible of the onset of fear and anxiety. Considering previous similar infectious diseases outbreaks, such as Severe Acute Respiratory Syndrome (SARS) and Middle East Respiratory Syndrome (MERS), several studies have shown their role in the occurrence of psychological implication, apprehension and distress both in healthcare workers and in patients [22,23,24].

Italian healthcare is mostly provided by public providers with some private or private-public entities. General taxation finances healthcare system [25]. The LEA (legislation on essential levels of care) are guaranteed by the central government and national funds are allocated to the Regions through the National Health Plan. Public healthcare system provides about 5% of oral healthcare services and oral healthcare is guaranteed only for specific populations such as children, vulnerable people both medically compromised and those on low income and individuals who need dental healthcare in some urgent/emergency cases [26]. It could be stated that Italian dental care is mainly provided under private arrangements. Not surprisingly, the majority of the respondents used to go to a private dental office, in accordance with a previous study [26]. Previous data suggested that the access to dental care has declined because of the economic crisis, showing increasing social inequalities due to socioeconomic, financial and education levels [27,28]. Considering the given answers about the main reason to cancel a dental appointment, only 8.9% of the respondents indicated a lowered economic income. According to the MCA results, subjects from North Italy were more likely to postpone a dental visit because of the lowered income household due to the SARS-CoV-2 pandemic. This datum could be explained by the fact that Northern Regions of Italy were the ones most heavily affected by coronavirus pandemic. Moreover, in accordance with previous studies [25,26], people from South Italy would have more difficulties in undergoing or resuming dental treatments than people from northern areas of Italy. An explanation could be feeling uncomfortable with dental care access during an emergency. The results of the present study showed that respondents who used to attend low-cost dental offices were more likely to report that the SARS-CoV-2 pandemic had a negative effect on their future dental appointments than those who were used to attend either public structures or private ones. In subjects attending private dental offices, affiliated private dental clinics or public structures, SARS-CoV-2 did not have a negative effect on the willingness to undergo dental appointments. An explanation of such a result could lie in the attention addressed to patient needs. Overall, dental patients will experience the service to be of higher quality when dentist–patient relationships are based on a feeling of trust rather than on a managerial model. The high level of trust expressed by respondents in their dentists could explain the great tranquility in facing the planned visits. The great sense of trust of dental patients in Italy towards their dentists and towards the implemented cross-infection control measures could be explained by the successful application in dentistry of universal infection control policies. Clinicians have to deal with blood-borne viruses and respiratory pathogens every day, which represents the highest risks of cross-infection [29]. In accordance with a recent study [30], quality patient-centered care plays a crucial role in the relationship of trust between patients and dentists.

According to Cotrin et al. [21], in Brazil, the coronavirus pandemic influenced orthodontic appointments and patients’ anxiety: subjects that would not go or would go only in urgency/emergency at dental office presented a significantly higher level of anxiety than the ones willing to attend an orthodontic visit. Additionally, in the present study, among Italians, those who were strongly influenced by coronavirus contagion fear had a significant higher risk to cancel or postpone the appointment at the dentist than the ones that were not influenced by SARS-CoV-2. The most selected reason to cancel a dental appointment was the non-urgent nature of dental cures. It could be stated that control visits and professional oral hygiene procedures could be the ones most affected by the effects of SARS-CoV-2. Preventive dentistry plays a crucial role in the interception of decays and periodontal diseases, as well as more severe diseases such as oral cancer [31,32,33,34]. If on the one hand a lack in preventive dental visits is likely to happen, on the other, an increase in the incidence of these diseases could occur in the next months. As a consequence, an increase in dental appointments could be likely to happen too, in order to overcome such oral health problems. During emergencies, patient management could be affected, and new strategies aimed at providing oral health are required. In such conditions, instant messaging platforms could be efficiently used in order to perform healthcare consultancies and to manage patients remotely [35].

The present study has two major limitations. On the one hand, the use of a non-probabilistic sampling technique may limit the representativeness of the sample. However, the snowball sampling has proven to be an efficient tool for data collection on social media, and in particular on Facebook. Baltar and Brunet, Scott and Vigar-Eliis and Kang et al. have demonstrated that snowball sampling adopted on Facebook manages to provide higher response rates than traditional snowball sampling, because of the strong personal links that exist on the social network between the researcher and the respondents [15,36,37]. Moreover, the snowball sampling technique has the advantage of expanding the sample size and reduce cost and time of research [36]. On the other hand, the present study was not a population-based study. Nevertheless, the survey was able to reach almost all the Italian Regions and a population with a wide range of ages (Table 1).

Limits notwithstanding, the present study allowed to assess the impact of the coronavirus pandemic on the willingness of people from different Italian Regions to undergo and to resume dental appointments. Moreover, it was possible to define patients’ profiles on the basis of their socio-demographic and behavioral characteristics.

## 5. Conclusions

Natural pandemics such as Sars-CoV-2 one are responsible of the onset of anxiety and fear but also awareness among the population. Especially in patients from Southern Italy and in those with high educational level Sars-CoV-2, fear could be the main reason to postpone dental appointments. The latter may lead to an increase in the incidence of diseases that could occur in the next months, due to the lack in prevention. Despite the forecasted economic crisis caused by coronavirus pandemic, feelings of fear and anxiety generated by the spread of the virus will impact more than the lowered familiar income in the access in dental care. Further studies are needed in order to assess the real impact of the SARS-CoV-2 pandemic on oral health, dental care access and dental offices.

## Figures and Tables

**Table 1:** Demographic information of the respondents to the questionnaire (*n* = 1003).

Demographics	Modalities	*n* (%)
Age Group	18–24	177 (17.6%)
	25–34	325 (32.4%)
	35–44	154 (15.7%)
	45–54	182 (18.1%)
	55–64	122 (12.2%)
	65–74	26 (2.6%)
	Over 75	14 (1.4%)
Gender	Female	609 (60.7%)
	Male	394 (39.3%)
Region of Residency	North	120 (11.9%)
	Central	481 (48.0%)
	South	402 (38.1%)
Marital Status	Unmarried	545 (54.3%)
	Married	379 (37.8%)
	Separated	33 (3.3%)
	Divorced	31 (3.1%)
	Widowed	15 (1.5%)
Number of people are in the household	1	85 (8.5%)
	2	197 (19.6%)
	3	253 (25.3%)
	4	355 (35.4%)
	4+	113 (11.2%)
Educational level	Primary School	3 (0.3%)
	Middle School	49 (4.9%)
	High School	327 (32.6%)
	Bachelor’s Degree	141 (14.1%)
	Master’s Degree	343 (34.1%)
	PhD/Specialization School	140 (14.0%)
Employment status	Employed in a public company	193 (19.2%)
	Employed in a private company	237 (23.7%)
	Self Employed/Freelance/Entrepreneur	213 (21.2%)
	Housekeeper	29 (2.9%)
	Unemployed	60 (6.0%)
	Student	241 (24.0%)
	Retired	30 (3.0%)
Changes into the income of the household brought by coronavirus pandemic	No, income remained unchanged	460 (45.9%)
	Yes, income was reduced up to 20%	245 (24.4%)
	Yes, income was reduced between 20 and 50%	164 (16.3%)
	Yes, income was reduced over 50%	100 (10.0%)
	Yes, household income reduced to zero	29 (2.9%)
	Other/I don’t know	5 (0.5%)

**Table 2:** Respondents’ answers regarding their attitude and habits toward dental care.

Question	Modalities	*n* (%)
What is the main reason for your visits at the dentist?	I only go for emergencies	276 (27.5%)
	I go for control visits or regular oral hygiene appointments	614 (61.2%)
	I am following a treatment plan	86 (8.6%)
	I don’t go/I’ve never been to a dentist	27 (2.7%)
Do you have a trusted dentist?	Yes	866 (88%)
	No	118 (12%)
Which kind of dentist clinic are you used to go to?	Private	919 (93.2%)
	Public	27 (2.7%)
	Low Cost Chain	14 (1.4%)
	Affiliated Private Clinic	26 (2.6%)
Where is the clinic you go to?	In the same province and city I live in	632 (64.3%)
	In the same province I live in, but in a different city	252 (25.6%)
	In a different province but in the same region I live in	46 (4.7%)
	In a different region from the one I live in	49 (5%)
	Abroad	4 (0.4%)
Since how many years do you visit the same dentist?	Less than one year	122 (12.4%)
	Two–three years	156 (16%)
	More than three years	700 (71.5%)
How often do you go to the dentist?	In case of necessity	302 (30.8%)
	At least once a year	370 (37.7%)
	A couple of times a year	207 (21.1%)
	Three times or more per year	102 (10.4%)
From March 9th to May 4th, 2020, did you need to undergo oral care?	Yes	94 (9.4%)
	No	343 (34.1%)
Having undergone the oral care treatment, how do you evaluate the experience?	Positively	77 (82.8%)
	Negatively	4 (4.2)
	I do not know	10 (10.8%)
	Other	2 (2.2%)
Do you have any scheduled appointment in the next months?	Yes	243 (24.2%)
	No	760 (75.8%)
How will or would you behave if you have scheduled appointments at the dentist?	I would undergo oral care without any problem	592 (59.1%)
	I would undergo oral care with fear	227 (22.6%)
	I would postpone the appointment	170 (16.9%)
	I would cancel the appointment	14 (1.4%)
What do you think might be the main reason to cancel a scheduled oral care appointment?	Fear of contagion	152 (15.1%)
	Non urgent cures	598 (59.7%)
	Lowered income	89 (8.9%)
	I’d rather the number of new cases to decrease more	147 (14.6%)
	Other	17 (1.7%)
How much do you trust your dentist for what concerns sterilization and sanitization of tools and environment?	Not at all	9 (1.1%)
	A Little	19 (2.2%)
	Quite	121 (14.3%)
	A Lot	291 (34.4%)
	Extremely	406 (48%)
Did your dentist give you all the information on the sanitization procedures adopted in the dental office?	Yes	364 (44.1%)
	No	462 (55.9%)
Through which means of communication did you get the information? You can select more than one option.	Phone Call	249 (33.5%)
	Talk	101 (13.6%)
	Message	97 (13%)
	Email	50 (6.7%)
	Social Network	68 (9.1%)
	Other	348 (46%)
Will you look for further information to feel safer with concern to access to oral care?	No, I feel safe and I won’t look for other information	309 (37.3%)
	Yes, I will directly call the clinic or the dentist	424 (51.1%)
	Yes, I will browse the website of the clinic	33 (3.9%)
	Yes, I will browse websites specialized in medicine	20 (2.4%)
	Yes, I will ask for information on social media	5 (0.6%)
	Yes, I will get informed through friends and family	11 (1.3%)
	Other	29 (3.4%)

**Table 3:** Odds ratio estimates of variables associated to the influence of SARS-CoV-2 on upcoming dentist appointments.

Independent Variable	Odds Ratio	95% C.I.		*p*-Value
Contagion Fear: “Yes” Vs “No”	1.59	1.1	2.29	0.013
Phase 2 Visit Behavior:				
attendance with fear vs postpone or cancel	0.51	0.32	0.8	0.003
attendance without fear vs postpone or cancel	0.25	0.16	0.37	<0.001
Phase 2 Postpone reason:				
fear vs lower income	3.12	1.44	7.38	0.006
decrease vs lower income	3.55	1.62	8.44	0.002
no urgency vs lower income	2.23	1.11	4.97	0.035
Dental Attendance:				
private vs low cost	0.21	0.06	0.69	0.012
public vs low cost	0.15	0.04	0.57	0.007
Education level: Low/Medium vs High	0.67	0.48	0.94	0.021

## Supplementary Materials

The following are available online at https://www.mdpi.com/1660-4601/17/19/6977/s1, Table S1: Questionnaire used in order to analyze the Italian patient’s anxiety, concerns or willingness to undergo dental care treatments after SARS-CoV-2. This is the English version of the original questionnaire in Italian language. Table S2: Creation and definition of new variables used for statistical analysis.

## References

[1] Report of the WHO-China Joint Mission on Coronavirus Disease 2019 (COVID-19). https://www.who.int/publications-detail/report-of-the-who-china-joint-mission-on-coronavirus-disease-2019-.

[2] WHO Director-General’s Opening Remarks at the Media Briefing on COVID-19—11 March 2020. https://www.who.int/dg/speeches/detail/who-director-general-s-opening-remarks-at-the-media-briefing-on-covid-19---11-march-2020.

[3] Ministero Della Salute Covid-19, Situation in Italy. http://www.salute.gov.it/portale/nuovocoronavirus/dettaglioContenutiNuovoCoronavirus.jsp?lingua=english&id=5367&area=nuovoCoronavirus&menu=vuoto.

[4] Gazzetta Ufficiale https://www.gazzettaufficiale.it/eli/id/2020/03/09/20A01558/sg.

[5] Gazzetta Ufficiale https://www.gazzettaufficiale.it/eli/id/2020/03/11/20A01605/sg.

[6] Lucchese M., Pianta M. (2020). The Coming Coronavirus Crisis: What Can We Learn?. Intereconomics.

[7] Lee K., Roehrer E., Cummings E. (2017). Information Overload in Consumers of Health-Related Information: A Scoping Review Protocol. JBI Database Syst. Rev. Implement. Rep..

[8] Gao J., Zheng P., Jia Y., Chen H., Mao Y., Chen S., Wang Y., Fu H., Dai J. (2020). Mental Health Problems and Social Media Exposure during COVID-19 Outbreak. PLoS ONE.

[9] Peng X., Xu X., Li Y., Cheng L., Zhou X., Ren B. (2020). Transmission Routes of 2019-NCoV and Controls in Dental Practice. Int. J. Oral Sci..

[10] Meng L., Hua F., Bian Z. (2020). Coronavirus Disease 2019 (COVID-19): Emerging and Future Challenges for Dental and Oral Medicine. J. Dent. Res..

[11] Fallahi H.R., Keyhan S.O., Zandian D., Kim S.-G., Cheshmi B. (2020). Being a Front-Line Dentist during the Covid-19 Pandemic: A Literature Review. Maxillofac. Plast. Reconstr. Surg..

[12] Izzetti R., Nisi M., Gabriele M., Graziani F. (2020). COVID-19 Transmission in Dental Practice: Brief Review of Preventive Measures in Italy. J. Dent. Res..

[13] Kampf G., Todt D., Pfaender S., Steinmann E. (2020). Persistence of Coronaviruses on Inanimate Surfaces and Their Inactivation with Biocidal Agents. J. Hosp. Infect..

[14] Gazzetta Ufficiale https://www.gazzettaufficiale.it/eli/id/2020/04/27/20A02352/sg.

[15] Scott L., Vigar-Ellis D. (2014). Consumer Understanding, Perceptions and Behaviours with Regard to Environmentally Friendly Packaging in a Developing Nation. Int. J. Consum. Stud..

[16] R Core Team (2018). R: A Language and Environment for Statistical Computing.

[17] Consolo U., Bellini P., Bencivenni D., Iani C., Checchi V. (2020). Epidemiological Aspects and Psychological Reactions to COVID-19 of Dental Practitioners in the Northern Italy Districts of Modena and Reggio Emilia. Int. J. Environ. Res. Public Health.

[18] Ahmed M.A., Jouhar R., Ahmed N., Adnan S., Aftab M., Zafar M.S., Khurshid Z. (2020). Fear and Practice Modifications among Dentists to Combat Novel Coronavirus Disease (COVID-19) Outbreak. Int. J. Environ. Res. Public Health.

[19] Shacham M., Hamama-Raz Y., Kolerman R., Mijiritsky O., Ben-Ezra M., Mijiritsky E. (2020). COVID-19 Factors and Psychological Factors Associated with Elevated Psychological Distress among Dentists and Dental Hygienists in Israel. Int. J. Environ. Res. Public Health.

[20] De Stefani A., Bruno G., Mutinelli S., Gracco A. (2020). COVID-19 Outbreak Perception in Italian Dentists. Int. J. Environ. Res. Public Health.

[21] Cotrin P.P., Peloso R.M., Oliveira R.C., Oliveira R.C.G., Pini N.I.P., Valarelli F.P., Freitas K.M.S. (2020). Impact of Coronavirus Pandemic in Appointments and Anxiety/Concerns of Patients Regarding Orthodontic Treatment. Orthod. Craniofac. Res..

[22] McAlonan G.M., Lee A.M., Cheung V., Cheung C., Tsang K.W.T., Sham P.C., Chua S.E., Wong J.G.W.S. (2007). Immediate and Sustained Psychological Impact of an Emerging Infectious Disease Outbreak on Health Care Workers. Can. J. Psychiatry Rev. Can. Psychiatr..

[23] Yip H.K., Tsang P.C.S., Samaranayake L.P., Li A.H.P. (2007). Knowledge of and Attitudes toward Severe Acute Respiratory Syndrome among a Cohort of Dental Patients in Hong Kong Following a Major Local Outbreak. Community Dent. Health.

[24] Ashok N., Rodrigues J.C., Azouni K., Darwish S., Abuderman A., Alkaabba A.A.F., Tarakji B. (2016). Knowledge and Apprehension of Dental Patients about MERS-A Questionnaire Survey. J. Clin. Diagn. Res. JCDR.

[25] Ferre F., de Belvis A.G., Valerio L., Longhi S., Lazzari A., Fattore G., Ricciardi W., Maresso A. (2014). Italy: Health System Review. Health Syst. Transit..

[26] Bindi M., Paganelli C., Eaton K.A., Widström E. (2017). The Healthcare System and the Provision of Oral Healthcare in European Union Member States. Part 8: Italy. Br. Dent. J..

[27] Tchicaya A., Lorentz N. (2014). Socioeconomic Inequalities in the Non-Use of Dental Care in Europe. Int. J. Equity Health.

[28] Elstad J.I. (2017). Dental Care Coverage and Income-Related Inequalities in Foregone Dental Care in Europe during the Great Recession. Community Dent. Oral Epidemiol..

[29] Jakubovics N., Greenwood M., Meechan J.G. (2014). General Medicine and Surgery for Dental Practitioners: Part 4. Infections and Infection Control. Br. Dent. J..

[30] Song Y., Luzzi L., Brennan D.S. (2020). Trust in Dentist-Patient Relationships: Mapping the Relevant Concepts. Eur. J. Oral Sci..

[31] Axelsson P., Nyström B., Lindhe J. (2004). The Long-Term Effect of a Plaque Control Program on Tooth Mortality, Caries and Periodontal Disease in Adults. Results after 30 Years of Maintenance. J. Clin. Periodontol..

[32] Petersen P.E. (2005). Sociobehavioural Risk Factors in Dental Caries—International Perspectives. Community Dent. Oral Epidemiol..

[33] Mascitti M., Orsini G., Tosco V., Monterubbianesi R., Balercia A., Putignano A., Procaccini M., Santarelli A. (2018). An Overview on Current Non-Invasive Diagnostic Devices in Oral Oncology. Front. Physiol..

[34] Varela-Centelles P., Diz-Iglesias P., Estany-Gestal A., Blanco-Hortas A., Bugarín-González R., Seoane-Romero J.M. (2020). Regular Dental Attendance and Periodontal Health Knowledge: A Cross-Sectional Survey. Oral Dis..

[35] Maspero C., Abate A., Cavagnetto D., El Morsi M., Fama A., Farronato M. (2020). Available Technologies, Applications and Benefits of Teleorthodontics. A Literature Review and Possible Applications during the COVID-19 Pandemic. J. Clin. Med..

[36] Baltar F., Brunet I. (2012). Social Research 2.0: Virtual Snowball Sampling Method Using Facebook. Internet Res..

[37] Kang J., Tang L.R., Fiore A.M. (2015). Restaurant Brand Pages on Facebook: Do Active Member Participation and Monetary Sales Promotions Matter?. Int. J. Contemp. Hosp. Manag..

