# Supplementary material for: Italian Response to Coronavirus Pandemic in Dental Care Access: The DeCADE Study
Source: Int J Environ Res Public Health. 2020 Sep 24;17(19):6977. doi: 10.3390/ijerph17196977 (PMC7579054; doi:10.3390/ijerph17196977)
Supplement: Supplementary file 1 [file ijerph-17-06977-s001.pdf]

Table S1. Questionnaire used in order to analyze the Italian patient's anxiety, concerns or willingness to undergo dental care treatments after SARS-CoV-2. This is the English version of the original questionnaire in Italian language.

Questions	Modalities
1. Select your age group	18-24
	25-34
	35-44
	45-54
	55-64
	65-74
	Over 75
2. Gender	Female
	Male
3. Region of Residency	Open Answer
4. Marital Status	Unmarried
	Married
	Separated
	Divorced
	Widowed
5. Including yourself, how many people are in your household?	1
	2
	3
	4
	4+
6. What is your educational level?	Primary School
	Middle School
	High School
	Bachelor's degree
	Master's degree
	PhD/Specialization School
7. What is your current employment status?	Employed in a public company
	Employed in a private company
	Self Employed/Freelance
	Entrepreneur
	Housekeeper
	Unemployed
	Student
	Retired
8. Did coronavirus pandemic reduce the income of your household?	No, income remained unchanged.
	Yes, income was reduced up to 20%
	Yes, income was reduced between 20 and 50%
	Yes, income was reduced over 50%
	Yes, household income reduced to zero
9. How much do you fear being infected by SARS-CoV-2?	Not at all
	A Little
	Quite
	A Lot
	Extremely
10. How much do you fear SARS-CoV-2 could infect your dearest ones?	Not at all
	A Little
	Quite
	A Lot

	Extremely
11. How much do you feel comfortable when you go out from your home?	Not at all
	A Little
	Quite
	A Lot
	Extremely
12. With respect to the modalities of contagion from coronavirus…	I feel anxious
	I feel quiet, it is enough to respect the safety measures
	I think the virus has lost its effect, it is less aggressive and contagious than before
13. How much do you think that fear of being infected might generate or has generated collective hysteria?	Not at all
	A Little
	Quite
	A Lot
	Extremely
14. Further than information received from TVs and newspapers, did you look for more information on Covid-19?	Yes
	No
15. Through which channels did you get informed on Covid-19? You can select more than one option.	Newspapers/Online newspapers
	TV/Radio
	Social Media (Facebook, Instagram, YouTube…)
	Blog/Forum
	Journals or Websites of Medicine, Health, Wellness
	Family Doctor/Other Doctors/Chemist
	Other

16. How much do you think these channels are reliable to get information on Covid-19? Assign a value from 1 to 5, where 1 is not at all and 5 is Extremely.

	1	2	3	4	5
Newspapers/Online newspapers					
TV/Radio					
Social Media (Facebook, Instagram, YouTube…)					
Blog/Forum					
Journals or Websites of Medicine, Health, Wellness					
Family Doctor/Other Doctors/Chemist					
Friends and Relatives					

17. How much do you think you are informed about the modalities of contagion from SARS-CoV-2?	Not at all
	A Little
	Quite
	A Lot
	Extremely
18. How much do you fear a new diffusion of coronavirus after the reopening of activities?	Not at all
	A Little
	Quite
	A Lot
	Extremely
19. How much do you think the measures adopted to limit contagion are effective?	Not at all
	A Little
	Quite
	A Lot
	Extremely
	At least 1 meter

20. What is the safety distance between two people who are NOT wearing a face mask?	2 meters
	More than 2 meters
21. What is the safety distance between two people who are both wearing a face mask?	At least 1 meter
	2 meters
	More than 2 meters
	The safety distance is not needed
22. If two persons are physically close and just one of them is wearing a face mask, is there a risk of infection?	Yes
	No
	It depends on the kind of face mask, some of them protect from contagion and at the same time don't allow to infect others
	I don't know
23. During Phase 2, do you think you will resume the activities you left behind at the beginning of the emergency?	As before the crisis, with no fear
	As before the crisis, with fear
	Less than before, with no fear
	Less than before and with fear
	With fear
	I won't resume
24. What is the main reason for your visits at the dentist?	I only go for emergencies
	I go for control visits or regular oral hygiene appointments
	I am following a treatment plan
	I don't go / I've never been to a dentist → if so, you can skip directly to question 30
25. Do you have a trusted dentist?	Yes
	No
26. Which kind of dentist clinic are you used to go to?	Private
	Public
	Low Cost Chain
	Affiliated Private Clinic
27. Where is the clinic you go to?	In the same Province and city I live in
	In the same Province I live in, but in a different city
	In a different Province but in the same Region I live in
	In a different Region from the one I live in
	Abroad
28. Since how many years do you visit the same dentist?	Less than 1 year
	2-3 years
	More than 3 years
29. How often do you go to the dentist?	In case of necessity
	At least once a year
	A couple of times a year
	Three times or more per year
30. From March 9th to May 4th, 2020, did you need to undergo oral care?	Yes
	No→ if so, you can skip directly to question 34
31. What was your behavior?	I underwent without any problem
	I underwent with fear
	I postponed them to a date to be determined
	The problem was solved telematically
	The Clinic postponed or cancelled the appointment

	The Dentist was wearing Personal Protective Equipment
32. How much did these measures make you feel safe? You can choose more than one option.	I could smell the disinfectant
	I was informed about the precautionary measures adopted
	I looked for information on various channels
	I fully trust my dentist
	I was not feeling safe
33. Having undergone the oral care treatment, how do you evaluate the experience?	Positively
	Negatively
	I do not know
	Other
34. Do you have any scheduled appointment in the next months?	Yes
	No
35. How will or would you behave if you have scheduled appointments at the dentist?	I would undergo oral care without any problem
	I would undergo oral care with fear
	I would postpone the appointment
	I would cancel the appointment
36. What do you think might be the main reason to cancel a scheduled oral care appointment?	Fear of contagion
	Non urgent cures
	Lowered income
	I'd rather the number of new cases to decrease more
	Other
37. During Phase 2, how comfortable would you feel in starting/going on with oral care?	Not at all
	A Little
	Quite
	A Lot
	Extremely
38. During Phase 2, how much do you think the risk of contagion could affect your next appointments?	Not at all
	A Little
	Quite
	A Lot
	Extremely
39. How urgent do you think it is urgent to resume with the cures you stopped?	Not at all
	A Little
	Quite
	A Lot
	Extremely
40. How much do you trust your dentist for what concerns sterilization and sanitization of tools and environment?	Not at all
	A Little
	Quite
	A Lot
	Extremely
41. Did your dentist give you all the information on the sanitization procedures adopted in the dental office?	Yes
	No
42. Through which means of communication did you get the information? You can select more than one option.	Phone Call
	Talk
	Message
	Email
	Social Network
	I didn't receive any information
	Other

43. Will you look for further information to feel safer with concern to access to oral care?	No, I feel safe and I won't look for other information
	Yes, I will directly call the Clinic or the dentist
	Yes, I will browse the website of the Clinic
	Yes, I will browse websites specialized in medicine
	Yes, I will ask for information on social media
	Yes, I will get informed through friends and family
	Other

Table S2. Reclassification of variables used for the analysis.

New Variable	Answers	Variable modality
"Educational level" Question number 6 Table S1	Primary School	Low/Medium
	Middle School	Low/Medium
	High School	Low/Medium
	Bachelor's Degree	High
	Master's Degree	High
	PhD/Specialization School	High
"Income reduction" Question number 8 Table S1	No, income remained unchanged.	< 50%
	Yes, income was reduced up to 20%	< 50%
	Yes, income was reduced between 20 and 50%	< 50%
	Yes, income was reduced over 50%	≥ 50%
	Yes, household income reduced to zero	≥ 50%
"Contagion fear" Question number 9 Table S1	≤ 3	No
	> 3	Yes
"Feeling comfortable when going out of home" Question number 11 Table S1	≤ 3	No
	> 3	Yes
"Fear of new spread of SARS-CoV-2" Question number 24 Table S1	I only go for emergencies	Regular visit
	I go for control visits or regular oral hygiene appointments	Regular visit
	I am following a treatment plan	Regular visit
	I don't go / I've never been to a dentist	Urgent care
"Dental attendance" Question number 26 Table S1	Private	Private
	Public	Public
	Low Cost Chain	Low cost
	Affiliated Private Clinic	Public
"Phase 2 Dental visit behavior" Question number 35 Table S1	I would undergo oral care without any problem	Attendance without fear
	I would undergo oral care with fear	Attendance with fear
	I would postpone the appointment	Postpone or cancel
	I would cancel the appointment	Postpone or cancel
"Feeling comfortable to attend dental visit" Question number 37 Table S1	≤ 3	No
	> 3	Yes
"Impact of contagion risk on dental visit" Question number 38 Table S1	≤ 3	No
	> 3	Yes
"Trusting dentist on sanitization procedures" Question number 40 Table S1	≤ 3	No
	> 3	Yes
"Dentist information on sanitization procedures" Question number 41 Table S1	≤ 3	No
	> 3	Yes
